# Nursing and midwifery regulation and HIV scale-up: establishing a baseline in east, central and southern Africa

**DOI:** 10.7448/IAS.16.1.18051

**Published:** 2013-03-25

**Authors:** Carey F McCarthy, Joachim Voss, Andre R Verani, Peggy Vidot, Marla E Salmon, Patricia L Riley

**Affiliations:** 1 Division of Global HIV/AIDS Center for Global Health, Centers for Disease Control and Prevention, Atlanta, GA, USA; 2 School of Nursing and Department of Global Health, University of Washington, Seattle, WA, USA; 3 Social Transformation Programmes Division, Commonwealth Secretariat, London, UK; 4 Evans School of Public Affairs, University of Washington, Seattle,WA, USA

**Keywords:** human resources for health, task shifting, task sharing, HIV, AIDS, scale-up, pre-service, nurse-initiated ART

## Abstract

**Introduction:**

Shifting HIV treatment tasks from physicians to nurses and midwives is essential to scaling-up HIV services in sub-Saharan Africa. Updating nursing and midwifery regulations to include task shifting and pre-service education reform can help facilitate reaching new HIV targets. Donor-supported initiatives to update nursing and midwifery regulations are increasing. However, there are gaps in our knowledge of current practice and education regulations and a lack of information to target and implement regulation strengthening efforts. We conducted a survey of national nursing and midwifery councils to describe current nursing and midwifery regulations in 13 African countries.

**Methods:**

A 30-item survey was administered to a convenience sample of 13 national nursing and midwifery regulatory body leaders in attendance at the PEPFAR-supported African Health Profession Regulatory Collaborative meeting in Nairobi, Kenya on 28 February, 2011. The survey contained questions on task shifting and regulations such as registration, licensure, scope of practice, pre-service education accreditation, continuing professional development and use of international guidelines. Survey data were analyzed to present country-level, comparative and regional findings.

**Results:**

Task shifting to nurses and midwives was reported in 11 of the 13 countries. Eight countries updated their scope of practice within the last five years; only one reported their regulations to reflect task shifting. Countries vary with regard to licensure, pre-service accreditation and continuing professional development regulations in place. There was no consistency in terms of what standards were used to design national practice and education regulations.

**Discussion:**

Many opportunities exist to assist countries to modernise regulations to incorporate important advancements from task shifting and pre-service reform. Appropriate, revised regulations can help sustain successful health workforce strategies and contribute to further scale-up HIV services and other global health priorities.

**Conclusions:**

This study provides fundamental information from which to articulate goals and to measure the impact of regulation strengthening efforts.

## Introduction

In much of sub-Saharan Africa, a severe shortage of health care workers and a disproportionately high burden of disease make it difficult to provide basic health services, much less meet ambitious new targets for combating HIV/AIDS [1–4]. Efforts by countries to respond to the HIV epidemic have highlighted the importance of the health workforce and have focused attention on the practice and education of health care workers [5–7]. Many global health initiatives, such as the President's Emergency Plan for AIDS Relief (PEPFAR), involve strategies to expand the capacity of the health workforce, such as strengthening health professional pre-service education and shifting the responsibility for delivering HIV services from physicians to mid-level health professionals (task shifting) [8–11]. Many HIV-related tasks previously assumed by physicians, such as diagnosing HIV infections, initiating anti-retroviral therapy (ART) and administering prevention of mother-to-child (PMTCT) regimens, are now carried out by nurses and midwives [5, 12–15]. There is wide agreement in the global health community that, in order to be sustainable, workforce strategies, such as pre-service strengthening and task shifting, should be incorporated into nationally endorsed health professional regulatory frameworks [9, 16–18]. Nursing and midwifery regulations that allow for and incorporate task shifting and pre-service strengthening can be instrumental in helping sub-Saharan African countries further expand HIV service delivery and reach new World AIDS Day targets [6, 10, 19–21].

Health professional regulation ensures the safety and quality of health professional practice and education [22–24]. Many countries in sub-Saharan Africa have a professional regulatory body, such as a nurses and midwives council, which issues the national regulations and standards for nursing and midwifery education and practice in their country (Table 1) [25, 26]. While regional and global leadership in the field has helped advance nursing and midwifery governance in the region, keeping regulations on pace with dynamic national and international health priorities has been challenging [27–29]. Global health donors and policy makers have encouraged revising key regulations, including licensing and registration, scopes of practice and pre-service education accreditation, to help ensure that advancements in service delivery from task shifting and pre-service reform are sustained [14, 30–32]. While the calls for revising regulatory frameworks mount, there are large gaps in our understanding of the current state of global health professional regulation and a lack of information from which to target and support regulation strengthening efforts [6, 9, 15, 17, 33].

**Table 1:** Common elements in nursing and midwifery legislation and regulation

Legislative and regulatory element	Explanation
Nurses and Midwives Act	Delineates authority of the regulatory body and functions of the registrar Authorises the regulatory body or ministry of health to issue more specific regulations (e.g., registration, licensing) to implement national nursing and midwifery legislation Defines the terms “nurse”, “midwife”, “nursing” and “midwifery”
Registration	Mandates that nurses and midwives register with the regulatory body Sets criteria and procedures for initial entry onto the register and criteria to maintain or lose registration status Allows for tracking the number and qualifications of nurses and midwives in the country, identification of deficits, planning and distribution.
Licensure	Sets criteria for initial issuance of a licence to practice as a nurse or midwife May mandate and administer an examination before a licence is issued Sets criteria for maintenance and renewal of licensure
Practice standards	Establishes what standard of care must be followed in the practice setting Sets the legal scope of practice each cadre must practice within Sets an expected code of conduct for delivery of care
Discipline and conduct standards	Prohibits and punishes illegal practice in order to protect professional titles Sets procedures for investigation of allegations Outlines sanctions for misconduct and process for appeals and reinstatement
Education standards	Describes different academic levels of programs and sets criteria for entering the programs Sets expected competencies for different levels of nurses and midwives Defines education standards and definitions of specialist and auxiliary personnel
Pre-service education accreditation	Sets criteria for formal recognition of a nursing and midwifery education program or institution by the regulatory body Sets requirements for faculty, lecturers and facility infrastructure Sets minimum standards for curricula, teaching methods and materials such as skills labs
Continuing professional development	Requires or authorises regulatory body to encourage or mandate continuing professional development (CPD) May mandate CPD for renewal of registration or licensure May require accreditation of CPD providers or accreditation of CPD content

From ref. 25 and 26.

**Figure 1:**
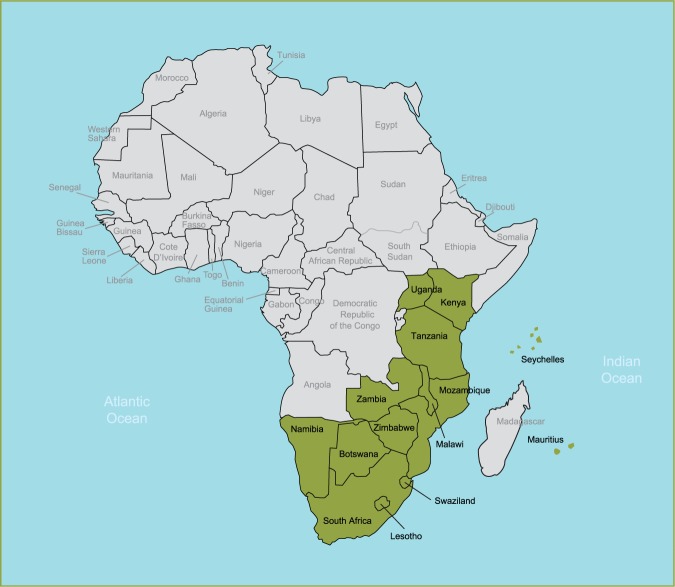
Map of countries participating in the ARC initiative, February 2011.

We conducted a survey of the leaders of national nursing and midwifery councils (called registrars) who are responsible for issuing and updating nursing and midwifery regulations in their country. The aim of the survey was to describe current nursing and midwifery practice and education regulations in 13 countries of east, central and southern Africa (ECSA). This is basic information necessary to assist the global health community and partner countries to better articulate and undertake regulation-strengthening efforts in the region. Furthermore, this information can serve as a baseline against which progress in advancing regulatory frameworks can be measured. The survey took place at a PEPFAR-supported conference, the African Health Profession Regulatory Collaborative for Nurses and Midwives (ARC), which convened nursing and midwifery leadership from 14 ECSA countries to discuss priorities and approaches to strengthening nursing and midwifery regulation in the region (Figure 1). The Registrar from each country was specifically invited to attend. Detailed information on the ARC initiative can be found elsewhere [34].

## Methods

A 30-item survey was administered on 28 February, 2011, to a convenience sample of ARC participants representing their national nursing and midwifery councils. The survey elicited information on key regulatory functions, such as registration and data collection, licensure, scopes of practice, accreditation of pre-service institutions, continuing professional development (CPD), standards used for setting regulations and task shifting from physicians to nurses and midwives. Questions were structured with yes/no choices and comment boxes, short answer and short essay questions. Study participants completed the written survey individually with no time limit. Surveys were conducted in English, as all of the countries, with the exception of Mozambique, were Anglophone. The Mozambican survey participant who did not speak English was provided an official translator to assist in completing the survey. All study materials were approved by the University of Washington Institutional Review Board and the US Centers for Disease Control and Prevention (CDC) Office of the Associate Director for Science.

## Results

Thirteen surveys were completed out of the 14 countries at ARC (the South Africa nursing council Registrar was invited but were unable to attend the meeting). Results from the surveys are provided below and divided into information on task shifting, registration and data collection, licensure, CPD, scopes of practice, pre-service accreditation and use of professional standards.

### Task shifting

Nursing and midwifery council registrars were asked if task shifting from physicians to nurses for HIV/AIDS and TB care occurred in their countries and whether practice and education regulations currently reflected task shifting. Eleven of the 13 registrars indicated that task shifting occurs, with some respondents stating that in their country, this type of task shifting was not yet “officially” endorsed. The registrars from Mauritius and Zimbabwe indicated that task shifting does not occur at all. Of the 11 countries in which task shifting is taking place, only Tanzania indicated that current regulations for nurses and midwives could allow for task shifting.

### Register of nurses and midwives

Survey participants were asked if they kept a national register of nurses and midwives and to indicate this data collected on the register (Table 2). All respondents indicated that there is a register maintained by the council (except for Mozambique which indicated that the register of health professionals is kept by the Ministry of Health). Each country with a register tracks “registered” professional nurses and midwives; nine councils collect data on the “enrolled” (i.e. vocational or practical) level of nurses and midwives. Eight councils also track nursing and midwifery students in pre-service education. The employment status and the professional specialty are collected by eight and six councils, respectively, but only two councils keep track of which nurses and midwives on the register are educators. In seven countries, the register is primarily electronic, while the remaining countries use a system that is still partly paper-based. All councils in this region require the nurse or midwife to maintain a current status on the register by “renewing” their registration or professional licence every one or three years.

**Table 2:** Nursing and midwifery council register information in east, central and southern Africa

Country*	Registered nurses and midwives	Enrolled nurses or midwives	Nursing specialty	Nurse educators	Nursing and midwifery students	Employment status	Type of register	Required renewal of status on register
Botswana	Yes	Yes	No	No	Yes	Yes	Paper & electronic	One year
Kenya	Yes	Yes	Yes	No	Yes	No	Paper & electronic	Three years
Lesotho	Yes	No	Yes	Yes	Yes	Yes	Paper & electronic	One year
Malawi	Yes	Yes	No	No	Yes	Yes	Electronic	One year
Mauritius	Yes	No	Yes	Yes	No	Yes	Electronic	One year
Namibia	Yes	Yes	No	No	Yes	Yes	Electronic	One year
Seychelles	Yes	No	No	No	No	No	Electronic	Three years
Swaziland	Yes	Yes	Yes	No	Yes	Yes	Electronic	One year
Tanzania	Yes	Yes	No	No	Yes	No	Electronic	Three years
Uganda	Yes	Yes	Yes	No	Yes	Yes	Paper & electronic	Three years
Zambia	Yes	Yes	No	No	No	Yes	Paper & electronic	One year
Zimbabwe	Yes	Yes	Yes	No	Yes	No	Electronic	One year

* In Mozambique, the registration of health professionals is done by the Ministry of Health.

### Licensure, continuing professional development and pre-service education accreditation

Registrars were queried about procedures for licensure, continuing professional development and accreditation of pre-service education (Table 3). Eleven ECSA councils issue licences to qualifying nurses and midwives. Five nursing and midwifery councils require a licensure examination to ensure that initial knowledge and competency standards are met; the remaining six councils issue licences upon proof of passing the final nursing education examination. Eight countries currently have national CPD programs for nurses and midwives; only three currently require proof of completing CPD in order to renew a professional licence. Almost all registrars responded that accreditation of pre-service education is the responsibility of the council yet only four indicated that accreditation status is renewed on a regular basis.

**Table 3:** Nursing and midwifery licensure, continuing professional development and accreditation of pre-service education in east, central and southern Africa

	Licensure		Continuing professional development (CPD)		Pre-service education accreditation	
			
Country	Licences are issued to nurses and midwives	Licensure exam required for nurses and midwives	CPD program in place	CPD required for licence renewal	Accreditation body	Renewal of accreditation status
Botswana	Yes	No	In design	N/A	No response provided	No response provided
Kenya	Yes	Yes	Yes	Yes	Nursing and midwifery council Commission for Higher Education	Requirement not established
Lesotho	Yes	Yes	In design	N/A	Nursing and midwifery council	Every two years
Malawi	Yes	Yes	Yes	Yes	Nursing and midwifery council	“Annually then periodically”
Mauritius	No	N/A	Yes	No	Nursing and midwifery council	Every two years
Mozambique	No	N/A	No	N/A	Ministry of Education Ministry of Health	“Regularly”
Namibia	Yes	Yes	Yes	Planned	National Qualification Authority	Requirement not established
Seychelles	Yes	No	Yes	Yes	None	N/A
Swaziland	Yes	No	In design	N/A	Nursing and midwifery council	No response provided
Tanzania	Yes	No	No	N/A	Nursing and midwifery council National Council for Technical Education Tanzania Commission for Education	Every two years
Uganda	Yes	Yes	Yes	Planned	Nursing and midwifery council	Requirement not established
Zambia	Yes	No	Yes	No	Nursing and midwifery council	Requirement not established
Zimbabwe	Yes	No	Yes	Yes	Nursing and midwifery council	“As necessary”

### Scope of practice

Nursing and midwifery council registrars were asked if they had an official scope of practice for nurses and midwives and how recently that scope of practice had been reviewed or revised (Table 4). All the surveyed countries indicated that there was an official scope of practice, with the exception of Mauritius and Mozambique where a broad scope of practice is defined for all civil service employees. Of the countries reporting a nursing and midwifery scope of practice, eight indicated that they had reviewed their scope of practice within the last five years, and three countries had reviewed them between 13 and 15 years ago.

**Table 4:** Nursing and midwifery scopes of practice in east, central and southern Africa

Country	Scope of practice exists in National Legislation, e.g., Nurses and Midwives Act	Most recent update of scope of practice
Botswana	Yes	“Regulations in the process of being gazetted”
Kenya	Yes	“Within last three years”
Lesotho	Yes	1998
Malawi	Yes	2008
Mauritius	No	N/A
Mozambique	No	N/A
Namibia	Yes	“Under review now”
Seychelles	Yes	“About to review Act”
Swaziland	Yes	2010
Tanzania	Yes	2010
Uganda	Yes	1996
Zambia	Yes	1997
Zimbabwe	Yes	2006

### Standards used to design practice and education regulations

Registrars were asked what standards were used as the basis for developing major regulations, such as the licensure examination, scope of practice, CPD program and pre-service education accreditation criteria (Table 5). Eleven countries reported using a combination of guidelines and standards from regional and international normative organizations (e.g., The East, Central and Southern College of Nursing, The International Council of Nurses) to design national regulations. It did not appear that countries used the same professional standards or guidelines in formulating their national regulations.

**Table 5:** Guidelines or standards used to design nursing and midwifery regulations in east, central and southern Africa

Country	Licensure examination	Scope of practice	Continuing professional development	Pre-service education accreditation
Botswana	N/A	ECSACON ICN ICM	Not yet decided	No response provided
Kenya	Ministry of Education Nursing and midwifery council	ECSACON ICN ICM	ICN ICM WHO	WHO
Lesotho	ECSACON ICN WHO	ECSACON ICN WHO	ECSACON ICN WHO	ECSACON ICN WHO
Malawi	ICN ICM Regional standards	ICN ICM Regional standards	ICM National standards	ICN Regional standards
Mauritius	N/A	ECSA ICN WHO	ECSA ICN	ICN
Mozambique	N/A	N/A	N/A	ICN National standards SANNAM WHO
Namibia	Nursing and midwifery council Training institutions	Nursing and midwifery council	Nursing and midwifery council Service providers	Nursing and midwifery council
Seychelles	N/A	International regulatory body	No response provided	N/A
Swaziland	N/A	ECSACON	ECSACON ICN	No response provided
Tanzania	N/A	ECSACON ICN	N/A	ICN
Uganda	Ministry of Education	Ministry of Health Nursing and midwifery council	Ministry of Health Nursing and midwifery council	Nursing and midwifery council
Zambia	N/A	Nurses and midwives act	Nursing and midwifery council	Nursing and midwifery council
Zimbabwe	N/A	ICN National standards	International guidelines National standards	International guidelines National standards

ECSACON, East, Central and Southern Africa College of Nursing; ICN, International Council of Nurses; ICM, International Confederation of Midwives; WHO, World Health Organization; SANNAM, South African Network of Nurses and Midwives.

## Discussion

Despite publication of the joint task shifting guidelines from the World Health Organization, PEPFAR and the United Nations Programme on HIV/AIDS in 2008, our understanding of how to adapt nursing and midwifery regulations to ensure sustainable task shifting and pre-service reform is weak. Establishing a baseline of nursing and midwifery regulations for sub-Saharan African countries can help identify strategic opportunities to assist countries in their efforts to advance nursing and midwifery practice and education. This study found that essential professional regulations, such as registration, licensure and pre-service accreditation were well established in most ECSA countries. Regulations addressing scope of practice, licensure examinations, CPD systems and accreditation renewal varied more widely and present potential areas for regulatory strengthening. For example, findings from this study indicate that even recently updated scopes of practice do not reflect the HIV services that nurses and midwives provide as a result of task shifting.

Only five of the ECSA countries surveyed require nurses and midwives to pass an examination by the nursing council prior to receiving a licence to practise. Licensure examinations can help ensure general competency but are also a means of testing and credentialing specialty knowledge. Training and licensing nurses for prescription of ART and management of HIV/AIDS patients has been implemented in some ECSA countries, most notably Botswana, South Africa and Zambia [21, 28, 35, 36], and could offer an effective method of expanding nurse-initiated HIV treatment and delivery of vital health services across sub-Saharan Africa. Many countries are also instituting or developing requirements for nurses and midwives to undergo CPD in order to renew their licence. CPD can help sustain important advancements in both pre-service education and the practice environment and ensure nurses and midwives who graduated prior to recent pre-service reforms are able to provide the same level of care as recent and future graduates. Countries could be supported in developing CPD modules that provide refresher training on ART and PMTCT regimens, teach new techniques for voluntary medical male circumcision and update practitioners on best practice guidelines.

For ECSA countries engaged in pre-service reform, accreditation of pre-service education is another important area for regulation strengthening. Accreditation criteria can be reviewed to ensure that nursing and midwifery programs include information on HIV and that PMTCT is integrated into maternal and child health curricula. Updated standards for education and accreditation that are applied across all institutions can increase the quality, relevancy and consistency of education in a country [30]. This study found a variety of national, regional and international guidelines and standards used to formulate accreditation standards in ECSA countries, suggesting that harmonisation in the region may be challenging. However, there is a precedent for regional coordination of nursing education standards and licensure examinations; Europe and the Caribbean provide recent examples [37, 38].

By describing the nursing and midwifery regulation currently in place in the ECSA region, this study enhances our understanding of how strengthened regulatory frameworks can help sustain successful workforce strategies and achieve global health priorities. Limitations of this study include a potential selection bias due to the use of convenience sample as opposed to a representative sample. The absence of data from South Africa, a leader in nursing and midwifery practice and education, results in an incomplete description of regulation in the region. Because this study focused solely on nursing and midwifery regulations primarily in countries with a British tradition of health professional education and legislation, the findings cannot be generalised beyond nursing and midwifery in the ECSA region. More specific country-level information is needed on what capacity or resources might be required to modernise regulatory frameworks in the region and how to best support regulatory bodies to carry out key revisions. Future research should investigate the extent to which task shifting takes place and its alignment with local regulations, pre-service education and health policy. Additional studies could also focus on effective ways to facilitate and objectively measure progress in regulatory strengthening.

## Conclusions

Reaching ambitious new targets for HIV/AIDS will require a continued commitment to strengthening the global health workforce. What is learned from the experiences of responding to HIV/AIDS can help improve the delivery of health services in a broad variety of health care settings. Integrating advancements to nursing and midwifery practice and education into national regulatory frameworks can help ensure the sustainability of these health workforce achievements. This study described the current state of nursing and midwifery regulations in 13 countries in sub-Saharan Africa and identified opportunities to strategically support countries in reviewing and revising regulations, such as scopes of practice, licensure, CPD and accreditation of pre-service institutions. Although we focused on ECSA, the findings have the potential to assist other countries and regions seeking to sustain successful workforce strategies through updated health professional regulation.
